# Coracoclavicular joint, an osteological study with clinical implications: a case report

**DOI:** 10.4076/1757-1626-2-8715

**Published:** 2009-08-07

**Authors:** Georgios Paraskevas, Marios-Efstathios Stavrakas, Alexandra Stoltidou

**Affiliations:** Department of Anatomy, Medical Faculty, Aristotle University of ThessalonikiThessalonikiGreece

## Abstract

**Introduction:**

The presence of an anomalous coracoclavicular joint was studied in a human male skeleton aged 73 years old from the Osteology Collection of our Department.

**Case presentation:**

We describe the exact morphology of this variation that is occasionally reported as an anatomical or radiological curiosity in the literature (0.55-21%).

**Conclusion:**

Although coracoclavicular diarthrosis is of no significance other than academic, it is important to recognize this variation and the clinical symptoms that may occur, as it is a cause of shoulder pain and arthritis in this or the adjacent sternoclavicular and acromioclavicular joint. Consequently, it is vital to apply the appropriate treatment.

## Introduction

Coracoclavicular joint is an anomalous diarthrosis formed between the conoid tubercle of the clavicle and the superior surface of the horizontal part of the coracoiod process of the scapula. It was first described in 1861 by Gruber. Since then, there have been reported a few cases of this variation, revealed on chest X-rays, dry skeletons or cadavers [1].

Though coracoclavicular diarthrosis is considered as a neglected structure without significance, its presence is useful to determining the aetiology of shoulder pain and its management in some instances. The presence of such a joint may cause in some cases shoulder pain radiating to the arm [2]. That relative rare anatomical structure with an incidence ranging from 0.55% to 21% [3,4] is stated that is more common in Asians than in Europeans or Africans [5].

The aim of the present study is to describe the morphology of a coracoclavicular joint found on macerated bones, as well as to report on the frequency and the correlation between this variant and any sexual, racial and tribal differences, and the possible clinical value of that variant.

## Case presentation

**Figure 1.**
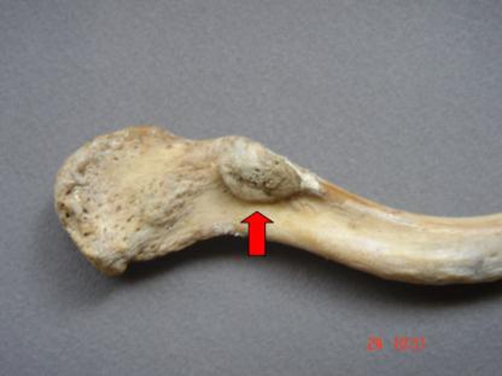
The clavicular facet (arrow) of the coracoclavicular joint, as seen on the inferior surface of the acromial end of a left clavicle.

**Figure 2.**
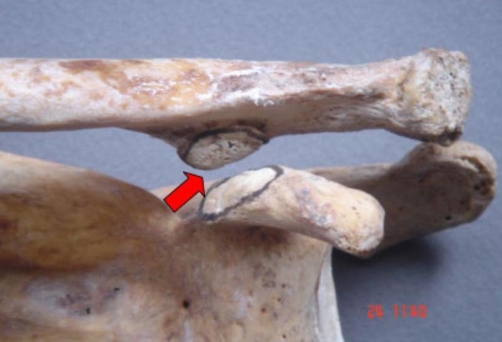
The articulation facets as they articulated in the coracoclavicular joint (arrow).

Our material was derived from the Osteological Collection of the Department of Anatomy of Medical Faculty of University of Thessaloniki. On the left clavicle and left scapula of a dried 73-year-old Caucasian male skeleton, two distinct anatomical features were found, in the area of the coracoclavicular syndesmosis. In particular, we came across an abnormal coracoclavicular joint at the left clavicle and left scapula. We noticed the existence of two outgrowths forming two articular facets, one on the coracoid process and the other on the inferior surface of the clavicle, near the conoid tubercle, 5 cm medial to the acromial edge. The noticed clavicular outgrowth was triangular in shape with the base at the clavicle and the apex directed downwards. The outer surface was oblique and smooth and it was in exact apposition with the chondral covered surface of the horizontal portion of the coracoid process. From the medical history of our specimen, there was a report of a diffuse left shoulder pain of unknown cause. The examined left clavicle and scapula didn’t appear any pathologic conditions or congenital abnormalities. The present study was approved by the University Research Ethic Committee. (Figure 1 and Figure 2).

## Discussion

The coracoclavicular joint is an anomalous, roundish joint, easily observable in primates. Its frequency ranges between 0.7% and 10% according to osteological studies or dissection [3,6] and between 0.55% and 21% according to radiological studies [1,5,6]. Coracoclavicular joint occurred unilaterally more frequently than bilaterally thus 0.49% versus 0.06% [1] and 5,02% versus 4,58% [7]. However, Cho et al [8] found the bilateral occurrence as higher at 8.8% of the 9,8% overall occurrence. The use of radiographs is of higher value in the study of the coracoclavicular joint than when dry bones are studied directly [1]. It is important to mention that only wet dissections can reveal with more accuracy the existence of cartilaginous facets, often regarded as evidence of a joint [9].

As far as sexual differences are concerned, it is found that this joint is more frequent in males than females in a proportion 11:1 [9]. However, most authors detected no statistically significant differences between the genders [1,3,7,8]. The variation seems to be more common in Asians than in other races [4,5,10]. This is contrary to the results presented by Nalla et al [7], who supported that both the white (10%) and black (9.4%) South African populations showed similar frequencies to those found in the Japanese and North-West Indian populations.

The development of arthritis at the coracoclavicular joint is not completely clear at the moment. According to Hall [2] any degenerative deformation of the coracoclavicular joint may cause cervicobrachial syndrome, because of the very close proximity to the brachial plexus. On the other hand, Cockshott’s study [5] revealed that coracoclavicular joint is subject to osteophytic marginal lipping, which is unable to create symptoms or disability. However, in some cases there have been described clinical symptoms attributed to coracoclavicular joint. Thus, these symptoms include shoulder-joint pain, radiating to the arm, the breast and neck, persisting during rest and increasing with exercise. The maximum intensity persisted at the site of the abnormal joint. Occasional symptoms included itching of the last four fingers, followed by transient paralysis of the hand [2]. In our case, the specimen during his life appeared a diffuse shoulder pain, presumably due to osteoarthritic changes of the coracoclavicular and acromioclavicular joint.

The possible complications of the existence of the anomalous joint that are presented in literature are humeral head fracture [11], cervicobrachial syndrome [12] and decrease in movement [2]. The etiology of the pain still seems obscure. Del Valle and Giordano [12] ascribed it to a sympathetic or plexal origin due to compression of microscopic nerves, which are relieved by removal of the anomalous joint. Other causes mentioned in literature are the degenerative changes of the joint and the close proximity to the brachial plexus, but they seem to be of little importance [2].

It has been suggested that the coracoclavicular joint is related to traumatic sequelae [13] and that the joint itself has a tendency to undergo arthritic changes [4]. Some authors have proposed that coracoclavicular joint may predispose to acromioclavicular or/and sternoclavicular degenerative changes. Possati [14] described the case of a workman who underwent acromioclavicular degenerative changes, while Kier et al [15] considered coracoclavicular joint as a predisposal factor for sternoclavicular degenerative changes. It is not known why coracoclavicular joint may predispose neighbouring joints to arthritic changes; nevertheless, it is plausible that the anomalous articulation plays a part in the stiffness of the acromioclavicular and sternoclavicular joints, reducing the capacity to absorb the different stresses applied on them [4].

Cho and Kang [8] correlated the appearance of coracoclavicular joint with the increase of age and raised the possibility that the joint may develop as a result of degenerative changes. Cockshott [5] proposed that the formation of coracoclavicular joint has genetical basis. On the contrary, Kaur and Jit [3] supported that this variant was not a result of any congenital malformation because neither of the 35 fetuses nor the 50 neonates studied showed the presence of the joint. They further reasoned that the formation of the joint later in life is conditioned more by genetic than by environmental factors.

Sexual and racial differences were not statistically significant. Nalla et al [7] found that individuals possessing the joint showed statistically significantly larger scapulae, longer clavicles and longer first ribs. Cho and Kang [8] supported that there is no correlation between the coracoclavicular joint and the size of the scapula, the clavicle length and the slope and heights of some coracoacromial arch elements. Gumina et al [4] observed no statistically significant differences between the mean length and the mean index of sinuosity of the anterior lateral curve of clavicles with and without coracoclavicular joint.
